# A Prognostic Microenvironment-Related Immune Signature *via* ESTIMATE (PROMISE Model) Predicts Overall Survival of Patients With Glioma

**DOI:** 10.3389/fonc.2020.580263

**Published:** 2020-12-07

**Authors:** Huaide Qiu, Yongqiang Li, Shupeng Cheng, Jiahui Li, Chuan He, Jianan Li

**Affiliations:** ^1^ Center of Rehabilitation Medicine, The First Affiliated Hospital of Nanjing Medical University, Nanjing, China; ^2^ Department of Rehabilitation Medicine, The Affiliated Jiangsu Shengze Hospital of Nanjing Medical University, Suzhou, China

**Keywords:** glioma, tumor microenvironment, immune signature, prognosis, biomarker

## Abstract

**Objective:**

In the development of immunotherapies in gliomas, the tumor microenvironment (TME) needs to be investigated. We aimed to construct a prognostic microenvironment-related immune signature *via* ESTIMATE (PROMISE model) for glioma.

**Methods:**

Stromal score (SS) and immune score (IS) were calculated *via* ESTIMATE for each glioma sample in the cancer genome atlas (TCGA), and differentially expressed genes (DEGs) were identified between high-score and low-score groups. Prognostic DEGs were selected *via* univariate Cox regression analysis. Using the lower-grcade glioma (LGG) data set in TCGA, we performed LASSO regression based on the prognostic DEGs and constructed a PROMISE model for glioma. The model was validated with survival analysis and the receiver operating characteristic (ROC) in TCGA glioma data sets (LGG, glioblastoma multiforme [GBM] and LGG+GBM) and Chinese glioma genome atlas (CGGA). A nomogram was developed to predict individual survival chances. Further, we explored the underlying mechanisms using gene set enrichment analysis (GSEA) and Cibersort analysis of tumor-infiltrating immune cells between risk groups as defined by the PROMISE model.

**Results:**

We obtained 220 upregulated DEGs and 42 downregulated DEGs in both high-IS and high-SS groups. The Cox regression highlighted 155 prognostic DEGs, out of which we selected 4 genes (CD86, ANXA1, C5AR1, and CD5) to construct a PROMISE model. The model stratifies glioma patients in TCGA as well as in CGGA with distinct survival outcome (P<0.05, Hazard ratio [HR]>1) and acceptable predictive accuracy (AUCs>0.6). With the nomogram, an individualized survival chance could be predicted intuitively with specific age, tumor grade, Isocitrate dehydrogenase (IDH) status, and the PROMISE risk score. ROC showed significant discrimination with the area under curves (AUCs) of 0.917 and 0.817 in TCGA and CGGA, respectively. GSEA between risk groups in both data sets were significantly enriched in multiple immune-related pathways. The Cibersort analysis highlighted four immune cells, i.e., CD 8 T cells, neutrophils, follicular helper T (Tfh) cells, and Natural killer (NK) cells.

**Conclusions:**

The PROMISE model can further stratify both LGG and GBM patients with distinct survival outcomes.These findings may help further our understanding of TME in gliomas and shed light on immunotherapies.

## Introduction

Gliomas are the most prevalent type of intracranial malignant neoplasms, accounting for 30% of tumors and 81% of malignancy in the brain (1). According to the 2016 World Health Organization (WHO) classification, malignant adult diffused gliomas consist of lower-grade glioma (grade II and III, LGG) and glioblastoma multiforme (grade IV, GBM) (2, 3). Besides, most of LGG progresses to GBM, which yielded a 5-year relative survival rate of 5% (4, 5). Standard treatments, including surgery, radiotherapy, and chemotherapy, have failed to address the poor survival outcomes of patients with glioma (6, 7). While immunotherapies are actively under investigation in the treatment of glioma, the tumor microenvironment (TME) related to immune response needs to be studied (6, 8).

TME has been extensively reported to alter the gene expression in tumor cells and subsequently prognosis of patients (9–11). Standalone therapies targeting the TME or in combination with traditional treatment have shown promise in improving clinical outcomes (12, 13). Therefore, systematic profiling of TME may shed light on prognostic stratification and innovative immunotherapies (14). An immune-related gene signature for GBM by Cheng W et al. indicated the significance of immune milieus in the prognosis of GBM patients; however, the signature was not evaluated with ROC analysis or compared with established classifications (15). Likewise, Deng X et al. (16) screened a total of 122 prognostic immune-related genes in LGG, which was only validated using Kaplan-Meier survival analysis without a specific cutoff value. Further, the number of genes stymies practical clinical implementation. Tian Y et al. (17) developed a stromal classifier based on local macrophage infiltration *via* Cibersort, but it was not validated in LGG and GBM independently. Plus, the predictive performance of the classifier was inferior to tumor grade, indicating the proposed classifier was not clinically-relevant. However, no other prognostic signature related to TME has been reported in glioma.

ESTIMATE, also known as “Estimation of Stromal and Immune cells in Malignant Tumors using Expression data”, is an algorithm for profiling TME. By analyzing specific gene expression, the algorithm infers the infiltration of stromal and immune cells and assesses tumor purity (18). Previous reports have applied the ESTIMATE to build prognostic models associated with TME in various malignancies, indicating the feasibility of the algorithm in the prediction of survival outcome (19–21). In the present study, we aimed to develop a Prognostic Microenvironment-related Immune Signature *via* ESTIMATE (PROMISE model) for glioma using expression profiles in The Cancer Genome Atlas (TCGA) and the Chinese Glioma Genome Atlas (CGGA) database. The signature was validated in LGG and GBM independently in both data sets, while the functional implication was explored with Gene Set Enrichment Analysis (GSEA) and immune cell analysis.

## Materials and Methods

### Acquisition Data from TCGA and CGGA Data Sets

From TCGA, The RNA-Seq data and clinical information of LGG and GBM samples were collected. Molecular subtype and treatment details were retrieved from Ceccarelli et al.’s work (22), while survival status and demographic information were accessed using the RTCGAclinical R package (version: 1.16.0). RNA-Seq data was measured as Fragments Per Kilobase of transcript per Million mapped reads (FPKM) and log2-based transformation. Then, the *sva* package (23) was utilized for the normalization of RNA expression profiles and to remove the batch effects between TCGA-LGG samples and TCGA-GBM samples. For external validation, normalized RNA-Seq data *via* RNA-Seq by Expectation Maximization (RSEM) (24) and clinical information were obtained from the CGGA data set. All data sets were downloaded on June 1st, 2020.

### Survival Analysis of Stromal Score (SS) and Immune Score (IS)

Stromal Score (SS) and Immune Score (IS) were calculated by the Estimation of Stromal and Immune cells in Malignant Tumors using Expression data (ESTIMATE) algorithm (18) in R software loaded with the *estimate* package. The scores represent the stromal and immune components in TME respectively. Glioma samples in TCGA were divided into high-score and low-score groups with the median of SS and IS respectively. Samples with positive values of survival time were used for survival analysis between groups with *survival* and *survminer* packages (25).

### DEGs Identification Based on SS and IS

The Linear Models for Microarray Data (LIMMA) package (26) in R software was used to replace replicates of probes with their average, while the Wilcoxon test was applied to extract DEGs between groups as defined by medians of SS and IS respectively. P < 0.05 and |log2FC| > 1 were set as the threshold for DEGs. The heatmap of the 100 DEGs with the most significant P values was plotted. Feature DEGs were identified as unanimously upregulated or downregulated DEGs in both the high-SS and high-IS groups. DEGs in independent cohorts (TCGA-LGG/TCGA-GBM) were also identified using the same methods.

### Functional Enrichment and Protein-Protein Interaction (PPI) Network Analysis

The *clusterProfiler* package (27) was utilized to perform the Gene Ontology (GO) terms and the Kyoto Encyclopedia of Genes and Genomes (KEGG) pathway enrichment analysis for feature DEGs. Three categories were included in the GO enrichment analysis, i.e., biological process (BP), cellular component (CC), and molecular function (MF); whereas, KEGG revealed enriched pathways related to these feature DEGs. Subsequently, the PPI network was constructed *via* Search Tool for the Retrieval of Interacting Genes (STRING) (28) to identify hub genes. Visualization of the network was performed in *Cytoscape 3.8.0* (29, 30).

### Development and Validation of a PROMISE Model in TCGA Data Sets

In the context of feature DEGs, univariate cox regression was conducted to identify prognostic DEGs, which was subsequently utilized in gene selection by the least absolute shrinkage and selection operator (LASSO) regression analysis (31) in the TCGA-LGG. And then we calculated the individualized risk score with coefficient-weighted gene expressions and constructed a PROMISE model with the following formula. Samples were divided into high-score and low-score groups based on the median risk score. Then the clinical relevance was validated using survival analysis between groups with the threshold of p < 0.05. The PROMISE model was then validated in the TCGA-GBM samples and the TCGA combined set with the same risk score formula and cutoff value. The receiver operating characteristic (ROC) analysis was performed, and we calculated the area under the curve (AUC) to evaluate the predictive accuracy (32) in the TCGA samples. Subsequently, a risk plot was presented that includes a heatmap presenting expression profiles of included genes, a distribution plot of risk scores based on the model, and a dot plot showing the survival status of patients in different risk groups.

Risk score=∑coefficient(i)×expression(i)

### Validation of the PROMISE Model in CGGA Data Sets

The PROMISE model was then validated using survival analysis in CGGA-LGG, CGGA-GBM, and CGGA data sets respectively. The Log-rank p-value was calculated while the Kaplan-Meier graph was plotted. P-values less than 0.05 were considered statistically significant. The prognostic performance was validated in the CGGA with ROC analysis as well as a risk plot.

### Development and Validation of a Nomogram

Clinicopathological factors were collected from TCGA data set and integrated with transcriptome profile derived from TCGA data set. The univariate and multivariate Cox regressions were conducted to determine whether the PROMISE model was independent of clinicopathological factors as well as to identify other independent prognostic factors. Based on independent prognostic factors, we formulated a nomogram to predict the survival probability of individual patients using methods reported in previous literature (33, 34). The ROC analysis for nomogram-based prediction was performed in both TCGA and CGGA data sets.

### Gene Set Enrichment Analysis

We performed Gene Set Enrichment Analysis (GSEA) between high-risk and low-risk groups as separated by the PROMISE model *via clusterProfiler* and *enrichplot* packages. The gseKEGG function was applied to identify the enriched pathways in KEGG.

### TME Analysis *via* Cibersort

To characterize the immune TME in different risk groups as defined by the PROMISE model, the Cibersort (35) method was adopted in our study. Immune cells with significant differences between risk groups were identified in TCGA data set and CGGA data set, respectively. We highlighted cells that were unanimously upregulated or downregulated in the high-risk groups in both data sets. Subsequently, Spearman correlation analysis was performed using the abundance of these cells and the PROMISE risk score.

## Results

### Preparation of Data Sets

**Figure 1:**
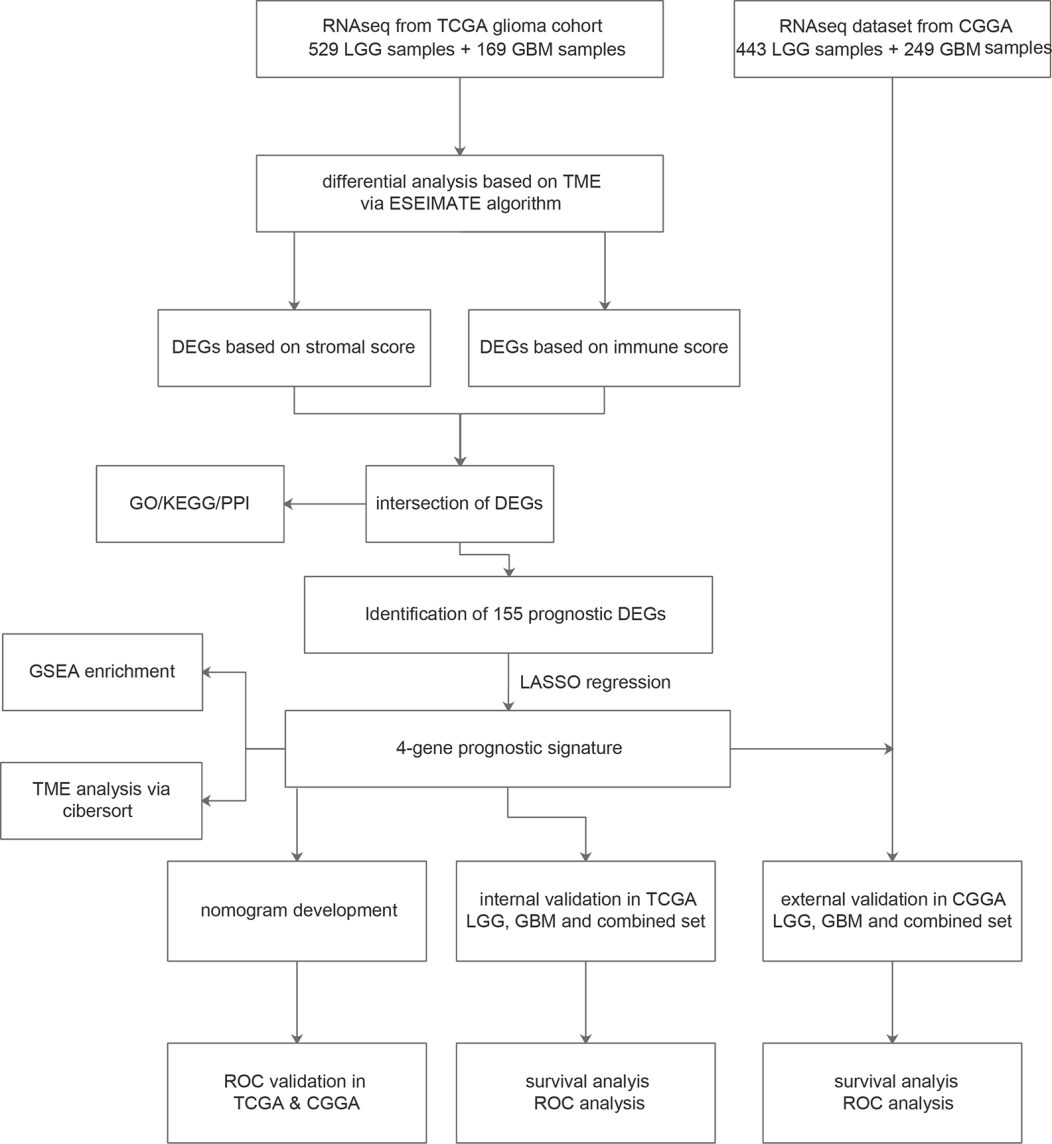
Flowchart of the study process.

The workflow of our study is shown in **Figure 1**. We obtained the RNA-Seq data and clinical information of 529 LGG samples and 169 GBM samples from TCGA, out of which 604 cases were recorded with positive values of survival time. Likewise, normalized RNA-Seq data and clinical information for 443 LGG samples and 249 GBM samples were collected from the CGGA. Among them, 657 cases had survival time>0. The distribution of cases concerning the clinicopathological factors was presented in **Table 1**.

**Table 1:** Clinicopathological factors of glioma patients included in the study.

Variables	TCGA (n = 604)	CGGA (n = 657)
Survival status		
Alive	421 (70%)	263 (40%)
Death	183 (30%)	394 (60%)
Follow-up time		
LGG(WHO II & III)	576 [334,1023.5]	1451 [656.5,2305]
GBM(WHO IV)	298 [154,454]	378 [221,768]
Sex		
Female	260 (43%)	283 (43%)
Male	344 (57%)	374 (57%)
Age		
>60	480 (79%)	588 (89.5%)
≤60	124 (21%)	68 (10.4%)
Not available		1 (0.1%)
Cancer type		
Primary	585 (97%)	404 (61%)
Recurrent	19 (3%)	253 (39%)
Grade		
WHO II	195 (32%)	172 (26%)
WHO III	206 (34%)	248 (38%)
WHO IV	157 (26%)	237 (36%)
Not available	46 (8%)	0
IDH.status		
Mutant	384 (64%)	333 (51%)
Wild type	212 (35%)	276 (42%)
Not available	8 (1%)	48 (7%)
X1p.19q.codeletion		
Code l	150 (25%)	137 (21%)
Non-code l	449 (74%)	454 (69%)
Not available	5 (0.01)	66 (10%)
MGMT.promoter.status		
Methylated	429 (71%)	304 (46%)
Unmethylated	141 (23%)	218 (33%)
Not available	34 (6%)	135 (21%)
Radiotherapy		
No	166 (27.5%)	131 (20%)
Yes	412 (68.2%)	501 (76%)
Not available	26 (4.3%)	25 (4%)
Chemotherapy		
No	190 (31.5%)	156 (24%)
Yes	381 (63.1%)	480 (73%)
Not available	33 (5.4%)	21 (3%)

### ESTIMATE Scores Were Correlated With the Survival of Glioma Patients

**Figure 2:**
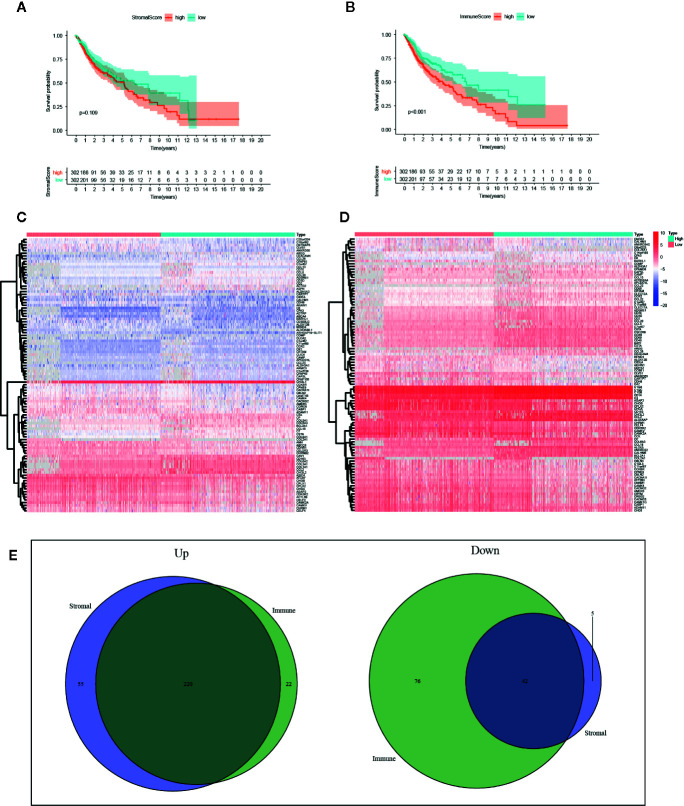
Identification of intersected DEGs based on stromal score (SS) and immune score (IS). **(A)** Survival analysis between high-SS group and low-SS groups. **(B)** Survival analysis between high-IS and low-IS groups. **(C)** Heatmap of the 100 DEGs with the most significant P values between the SS groups. **(D)** Heatmap of the 100 DEGs with the most significant P values between the IS groups. **(E)** Venn plots of the feature DEGs identified as unanimously upregulated or downregulated DEGs in both the high-SS and high-IS groups.

With the ESTIMATE algorithm, we calculated SS and IS for all these TCGA samples. To explore the potential association of survival rate in glioma patients with the stromal and immune components in TME, we classified these samples into high-score and low-score groups with the median scores. Survival analysis revealed that the survival time of samples with high SS was shorter than that of samples with low scores, yet no significant difference was detected (**Figure 2A**, p=0.109). The survival analysis presents the same trend between groups as separated by the median of IS with a statistical difference (**Figure 2B**, p<0.001).

### Identification of DEGs Based on SS and IS

According to the threshold, we identified DEGs based on SS or IS scores respectively. The heatmap of the 100 DEGs with the most significant P values was plotted in **Figures 2C, D**. In order to identify key DEGs on different TME, we obtained 220 unanimously upregulated DEGs and 42 unanimously downregulated DEGs in both high-IS and high-SS groups (**Figure 2E**). The subsequent analysis was based on these feature DEGs, which can be accessed in **Supplementary File S1**. In parallel, we also identified DEGs in TCGA-LGG and TCGA-GBM, respectively (**Supplementary Files S2** and **S3**).

### Enrichment Analyses of Feature DEGs and PPI Network Construction

**Figure 3:**
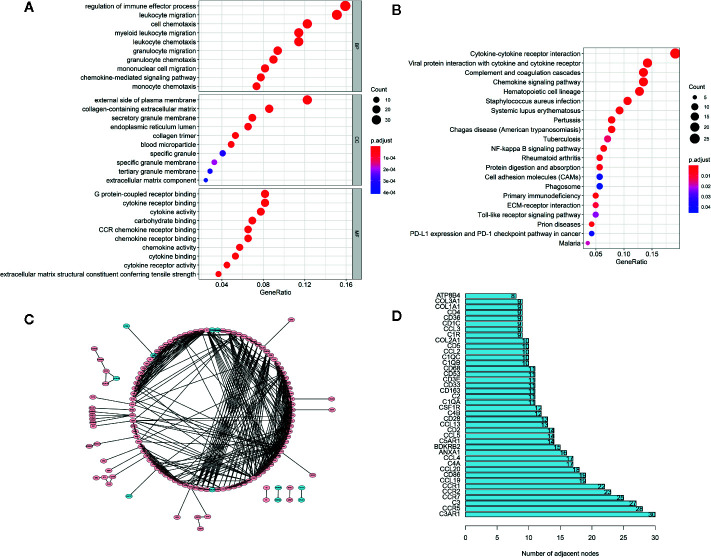
Functional Enrichment and PPI Network Analysis. **(A)** The top 30 significantly enriched GO terms. **(B)** The top most enriched KEGG pathways. **(C)** PPI network analysis. **(D)** Potential hub genes and their numbers of adjacent nodes.

The GO analysis (**Figure 3A**) demonstrated that these feature DEGs were significantly enriched the BP that includes regulation of immune effector process, leukocyte migration, cell chemotaxis, and so on. The CC analysis indicated that these feature DEGs were present on the external side of the plasma membrane, collagen-containing extracellular matrix, secretory granule membrane, etc. The feature DEGs were enriched in MF, such as G protein-coupled receptor binding, cytokine receptor binding, and cytokine activity. Furthermore, the KEGG analysis indicated that the genes were mainly involved in Cytokine-cytokine receptor interaction, which was closely related to the immune response (**Figure 3B**). Then we constructed a PPI network to further explore the interplay among the feature DEGs and obtained 40 hub genes with the most interactions (**Figures 3C, D** **)**.

### Development and Validation of a PROMISE Model in TCGA Data Set

**Figure 4:**
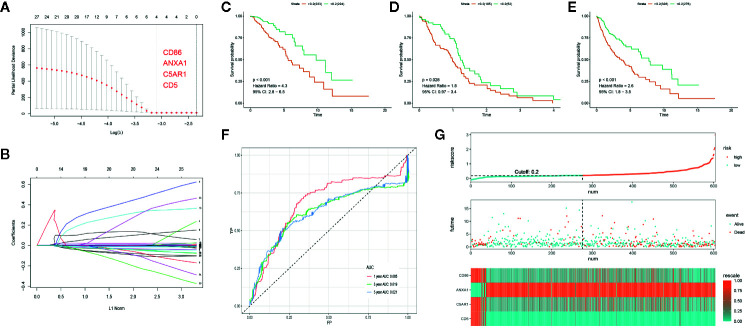
Development of the PROMISE model for TCGA-LGG and validation in TCGA-GBM and TCGA combined set. **(A, B)** Gene selection by the LASSO regression analysis. **(C)** Survival analysis between high-risk and low-risk groups in TCGA-LGG. **(D)** Survival analysis between high-risk and low-risk groups in TCGA-GBM. **(E)** Survival analysis between high-risk and low-risk groups in TCGA combined set. **(F)** ROC curve analysis of the PROMISE model in TCGA combined set. **(G)** Risk plot encompassing distribution of groups based on the PROMISE model in TCGA combined set.

Out of the 262 feature DEGs, a total of 155 prognostic DEGs were identified using univariate cox regression (**Supplementary File S4**). Based on these prognostic DEGs, we selected a total of 4 specific DEGs (CD86, ANXA1, C5AR1, and CD5) in the TCGA-LGG with Lasso regression (**Figures 4A, B**). After extracting the coefficient values, we calculated the PROMISE risk scores with coefficient-weighted expression levels of 4 hub genes with the following formula:

Risk score=CD86*0.013936078+ANXA1*0.004725055+C5AR1*0.004208545+CD5*0.348764054

LGG samples were divided into high-risk and low-risk groups based on the median risk score (0.20). Significant difference was shown between groups with survival analysis (**Figure 4D**, HR = 4.3, 95% CI = 2.8 - 6.5, p < 0.001). Subsequently, we calculated individualized risk scores in TCGA-GBM samples and divided them into high-risk and low-risk groups with the same cutoff value. A significant difference was also presented between groups with survival analysis in TCGA-GBM (**Figure 4C**, HR = 1.8, 95% CI = 0.97–3.4, p=0.028). Then we repeated the same validation method in the TCGA combined set and yielded distinct separation in survival outcomes with p<0.001(HR = 2.6, 95% CI = 1.8–3.5) (**Figure 4E**). ROC curve analysis of the PROMISE model achieved AUC of 0.685, 0.619, and 0.621 at 1-, 3-, and 5-year (**Figure 4F**). **Figure 4G** shows the risk plot in TCGA glioma cohort, including the distribution of risk score and accurate classification of survival outcomes across different risk groups with red dots being dead and blue ones being living cases. The expression levels of four hub genes demonstrated a trend of upregulation of ANXA1 along with downregulation of CD86, C5AR1, and CD5 in the high-risk group as classified by the PROMISE model (**Figure 4G**).

### Validation of the PROMISE Model in the CGGA

**Figure 5:**
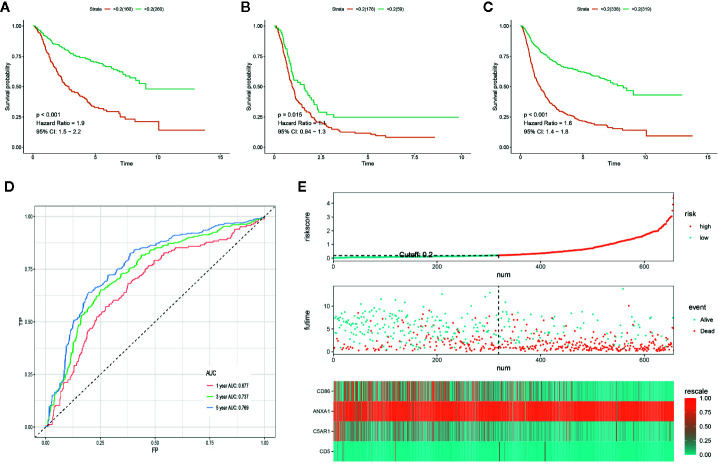
Validation of the PROMISE model in CGGA. **(A)** Survival analysis between high-risk and low-risk groups in CGGA-LGG. **(B)** Survival analysis between high-risk and low-risk groups in CGGA-GBM. **(C)** Survival analysis between high-risk and low-risk groups in CGGA combined set. **(D)** ROC curve analysis of the PROMISE model in CGGA combined set. **(E)** Risk plot encompassing distribution of groups based on the PROMISE model in CGGA combined set.

In order to determine if the PROMISE model was independent of data sets, we validated the model in the CGGA cohort. Survival analysis showed significant differences in CGGA-LGG, CGGA-GBM, and CGGA combined set with p<0.001, p=0.015, and p<0.001 respectively (**Figures 5A, B, C**). ROC curve analysis of the PROMISE model in CGGA Glioma samples achieved AUCs of 0.677, 0.737, and 0.769 at 1-, 3-, and 5-year (**Figure 5D**). Likewise, the risk plot of CGGA encompassing the distribution of the PROMISE risk score, survival status of cases as well as gene expression of 4 hub genes was presented with a similar expression pattern to TCGA data set (**Figure 5E**).

### Development and Validation of a Nomogram

**Figure 6:**
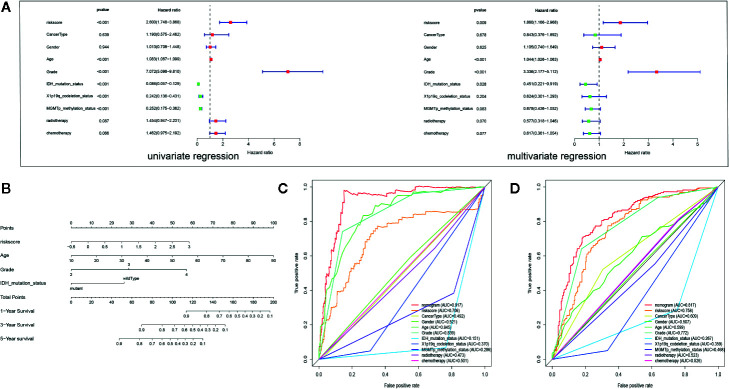
Development and validation of a Nomogram. **(A)** Univariate and multivariate Cox regression analysis for Glioma with clinicopathological factors in the TCGA data set. **(B)** The nomogram based on the independent prognostic factors. **(C)** ROC curve analysis of the all clinicopathological factors in TCGA data set. **(D)** ROC curve analysis of the all clinicopathological factors in CGGA data set.

Multivariate analysis revealed that the PROMISE model based on 4 hub genes could be an independent prognostic marker after clinical characteristics were adjusted. Likewise, age and tumor grade were also independent prognostic factors (**Figure 6A**). Based on the independent prognostic factors, we formulated a nomogram to predict the survival probability of individual samples (**Figure 6B**). Individualized survival chance could be predicted intuitively with specific age, tumor grade, Isocitrate dehydrogenase (IDH) mutation status, and the PROMISE risk score. ROC curve analysis showed adequate discrimination with an AUC of 0.917 and 0.817 in TCGA Glioma samples (**Figure 6C**) and CGGA Glioma samples (**Figure 6D**) respectively, which outperformed all clinicopathological factors including classifications of molecular subtype.

### Gene Set Enrichment Analysis

**Figure 7:**
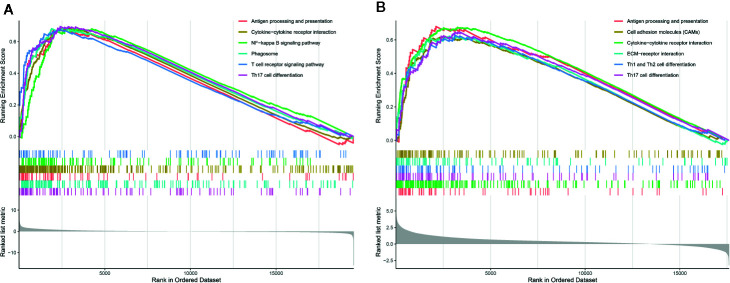
Gene Set Enrichment Analysis (GSEA) analysis. **(A)** GSEA of KEGG pathways in TCGA glioma samples between risk groups based on the PROMISE model. **(B)** GSEA in KEGG pathways in CGGA Glioma samples between risk groups based on the PROMISE model.

To explore the underlying mechanism, we performed GSEA between groups based on the PROMISE model to identify the enriched KEGG pathway. **Figure 7A** shows the results of the KEGG analysis in TCGA Glioma samples. Antigen processing and presentation, cytokine-cytokine receptor interaction, NF-kappa B signaling pathway, phagosome, T cell receptor signaling pathway, and Th17 cell differentiation were significantly enriched. In CGGA Glioma samples (**Figure 7B**), antigen processing and presentation, cell adhesion molecules, cytokine-cytokine receptor interaction, ECM- receptor interaction, cell differentiation of Th1, Th2, and Th17 were significantly enriched.

### TME Analysis *via* Cibersort

**Figure 8:**
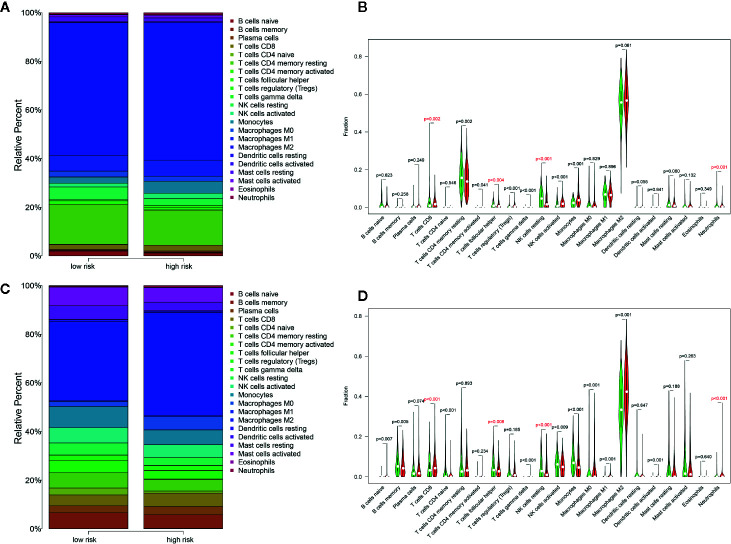
Distribution of immune cells between risk groups. **(A)** Proportions of immune cells based on risk groups in TCGA glioma samples. **(B)** Violin plot of the differentiation of immune cells between risk groups in TCGA glioma samples. **(C)** Proportions of immune cells based on risk groups in CGGA Glioma samples. **(D)** Violin plot of the differentiation of immune cells between risk groups in CGGA Glioma samples.

Distribution levels of 22 immune cells based on risk groups in TCGA Glioma samples and TCGA Glioma samples were shown in **Figures 8A, C**. Immune cells with significant differences between risk groups were shown in **Figures 8B, D** respectively. Cells that were unanimously upregulated or downregulated in both data sets were identified. The specific immune cells were marked in red. It is shown that T cells CD8 (p=0.002, p<0.001) and Neutrophils (p<0.001, p<0.001) level were significantly enriched in the high-score group while lower T cell follicular helper (p=0.004, p=0.008) and natural killer (NK) cells resting (p<0.001, p<0.001) were observed in high-risk groups in both TCGA and CGGA data sets. Spearman correlation analysis indicated that these cells were correlated with the PROMISE model in both data sets (**Supplementary File S5**).

## Discussions

Due to the biological diversity of glioma cells, multimodal treatments remained unsatisfactory with surgical resection, radiotherapy, and temozolomide (36). Stimulated by the progress in immunotherapies on other tumor types, researchers have been actively examining the effect of immunotherapies in gliomas (6). While pursuing novel immune-related therapeutic approaches in gliomas, the TME needs to be investigated. Biomarkers related to the TME may help predict treatment response and prognosis in patients with glioma.

In the present study, we selected 262 feature DEGs based on TME scores as defined by the ESTIMATE algorithm. Out of the 262 DEGs, we identified 155 prognostic DEGs, whereby we constructed a PROMISE model with 4 genes identified *via* LASSO regression. The PROMISE model was observed to be clinically relevant with distinct separation of survival rate between risk groups in patients with LGG, GBM as well as overall glioma in two independent cohorts (TCGA and CGGA). With a multivariate regression, we demonstrated that the PROMISE risk score could be an independent prognostic index after clinicopathological factors were adjusted. Subsequently, we selected independent prognostic factors and developed a nomogram that could intuitively predict individual survival probability. Further, the nomogram presents effective discrimination with AUCs that outperformed all clinicopathological factors in both data sets. Further, we explored the underlying mechanisms using GSEA and analysis of 22 types of tumor-infiltrating immune cells *via* Cibersort between risk groups as defined by the PROMISE model.

The PROMISE model highlighted 4 TME-related genes, i.e., CD86, ANXA1, C5AR1, and CD5. Annexin-1 (ANXA1) is a substrate for the epidermal growth factor receptor (EGFR) involved in cellular proliferation, apoptosis, and inflammatory response (37–39). Overexpression of ANXA1 was observed in astrocytoma in the immunohistochemical analysis (40); whereas, its prognostic value in glioma patients was reported in a previous cross-validated model study (41). CD86 (B7-2), one of the checkpoint proteins in antigen-presenting cells (APCs), interacts with CD28 and cytotoxic T lymphocyte antigen‐4 (CTLA‐4) receptors on T cells, thereby limiting T-cell activation and inducing immunoescape of cancers (42, 43). The expression of CD86 was associated with poor prognosis in myeloma (44) and leukemia (45). CA5R1 serves as the receptor of CA5, which was associated with inhibition of antitumor T-cell responses through the recruitment and/or activation of immunosuppressive cells. Therefore, poor prognosis was presented in cancer survivors with high expression of CA5R1 (46–49). CD5 was identified as a prognostic factor in large B-cell lymphoma with increased expression correlated to inferior survival (50). However, the expression of CD86, CA5R1, and CD5 seemed to be downregulated in the high-risk group, which could be due to the uniqueness of TME in the brain. The role of these genes in glioma patients is yet to be elucidated. Our study demonstrated the prognostic significance of the four genes associated with TME in glioma patients, indicating that these genes could be candidate targets for enhancing the therapeutic effects of immunotherapy.

To explore the potential mechanisms by which the PROMISE model classifies glioma patients with distinct survival outcomes, we performed GSEA to investigate the enriched pathways in the high-risk group. Enhanced activities in extensive immune-related pathways were revealed in both TCGA and CGGA data sets, i.e., antigen processing and presentation, cytokine, and its interaction with receptors, as well as Th17 cell differentiation. To further investigate the role of TME associated with the PROMISE model, we analyzed the estimations of 22 types of tumor-infiltrating immune cells *via* Cibersort analysis. The results demonstrated significant differences in immune cell abundance between risk groups in both data sets, which highlighted four immune cells in the high-risk group as defined by the PROMISE model. Among them, CD 8 T cells and neutrophils were upregulated, while the follicular helper T (Tfh) cells and Natural killer (NK) cells were downregulated. A murine model study showed that CD 8 T cells promoted immunoediting of immunogenic tumor clones by negative selection in the formation of gliomas, thereby facilitating tumor progression (51). Additionally, activation of CD 8 T cells results in a decreased expression of Interferon-γ (IFN-γ) and tumor necrosis factor-α (TNF-α), leading to inhibition of the antitumor response (52). By contrast, downregulated Tfh cells in the high-risk groups indicated the tumor-suppressive effect of Tfh cells. Although there have been no relevant studies on gliomas, studies regarding lymphomas and breast cancers have shown that Tfh cells can regulate B cell response, convert Treg-mediated immune suppression, and inhibit tumor growth *via* adaptive antitumor humoral responses (53, 54). NK cells can trigger tumor cell killing by binding their surface NKG2D receptors with upregulated ligands in glioma cells (55, 56). Our findings are consistent with the effects, indicating that NK cells kill glioma cells and improve prognosis. Neutrophils were observed to promote the proliferation and migration of glioblastoma-initiating cells (GICs), leading to tumor progression (57). In summary, the disproportion of immune cells in TME may have an important role in gliomas. Further experiments are required to investigate the individual effects and the cross-talk of these immune cells in TME.

Although numerous studies have examined gene signatures related to TME in patients with glioma, their efforts could not be translated to clinical practice due to vague cutoff values across different data sets (15–17), lack of comparisons with established classification (15, 16), inferior classification accuracy compared to tumor grade (17), and a relatively large number of genes (16). In contrast, our PROMISE model with four genes embedded used an identical cutoff value across different data sets (LGG and GBM in both TCGA and CGGA) and presented distinct survival rates between risk groups. Further, the nomogram constructed with clinicopathological factors yielded superior accuracy to established classifications. To our best knowledge, the PROMISE model in the present study has been the first prognostic signature derived from systematic profiling of immune microenvironment *via* ESTIMATE across different grade of glioma. It has shown a distinct separation of survival outcomes in glioma patients with adequate predictive performance, which could be translated to clinical settings as a prognostic biomarker. Therapeutics targeting genes embedded in the PROMISE model might yield promising results in the treatment of glioma. Although we validated the model in independent cohorts in this bioinformatics report, a lack of experimental validation is considered to be the major limitation. The biological functions of the four genes require further investigation in laboratory settings.

## Conclusions

In the present study, we identified a PROMISE model that could effectively classify glioma patients with distinct survival outcomes, for which the altered TME might be responsible. These findings may further our understanding of the TME and shed light on the development of novel prognostic biomarkers and therapeutic targets in gliomas.

## Data Availability Statement

All data used in the study are available in The Cancer Genome Atlas (TCGA) (https://portal.gdc.cancer.gov/) and Chinese Glioma Genome Atlas (CGGA) (http://www.cgga.org.cn).

## Author Contributions

HQ: Conceptualization, data curation, formal analysis, roles/writing—original draft, writing—review and editing. YL: Funding acquisition, investigation, methodology. SC and JHL: Roles/writing—original draft, writing—review and editing. CH and JAL: Funding acquisition, methodology, project administration, resources, supervision. All authors contributed to the article and approved the submitted version.

## Funding

This study was funded by Key project of Jiangsu Provincial Department of science and technology (BE2017007-5), the Nanjing Municipal Science and Technology Bureau (No. 2019060002), and the Introduced Project of Suzhou Clinical Medical Expert Team (SZYJTD201725).

## Conflict of Interest

The authors declare that the research was conducted in the absence of any commercial or financial relationships that could be construed as a potential conflict of interest.

## Supplementary Material

Supplementary File S1 Gene list of feature DEGs based on ESTIMATE in TCGA-glioma.

Supplementary File S2 Venn diagram of differential analysis based on ESTIMATE in independent cohorts.

Supplementary File S3 Gene list of DEGs based on ESTIMATE in independent cohorts.

Supplementary File S4 Prognostic DEGs identified using univariate cox regression.

Supplementary File S5 Correlation between the immune cell abundance and the PROMISE risk score. **(A–D)** Scatter plot of the Spearman correlation analysis in TCGA glioma samples. **(E–H)** Scatter plot of the Spearman correlation analysis in CGGA Glioma samples.
