# Supplementary figures and images for: A Prognostic Microenvironment-Related Immune Signature via ESTIMATE (PROMISE Model) Predicts Overall Survival of Patients With Glioma
Source: Front Oncol. 2020 Dec 7;10:580263. doi: 10.3389/fonc.2020.580263 (PMC7793983; doi:10.3389/fonc.2020.580263)

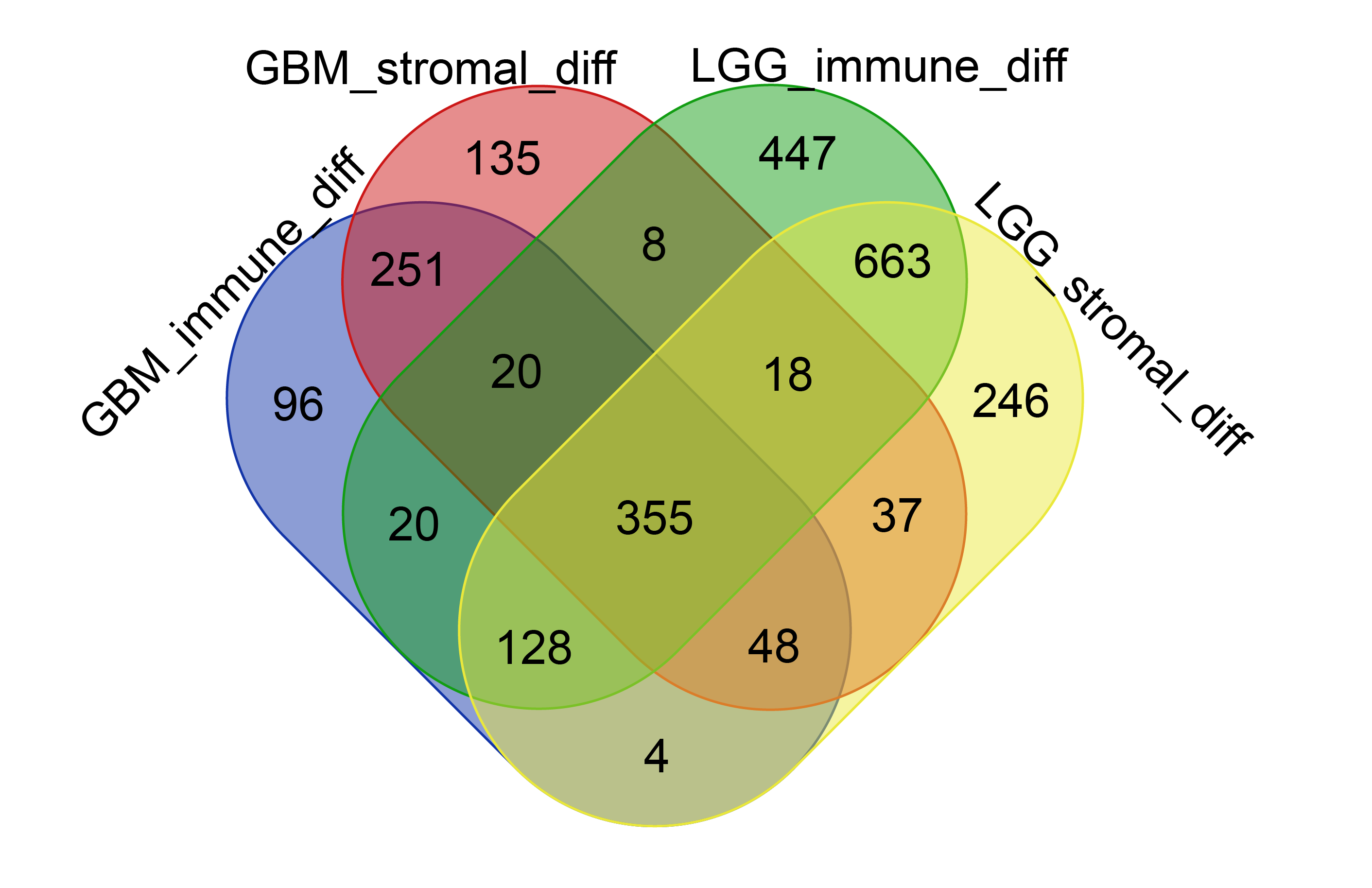

Supplement: Supplementary File S2 — Venn diagram of differential analysis based on ESTIMATE in independent cohorts. [file Image_1.tif]

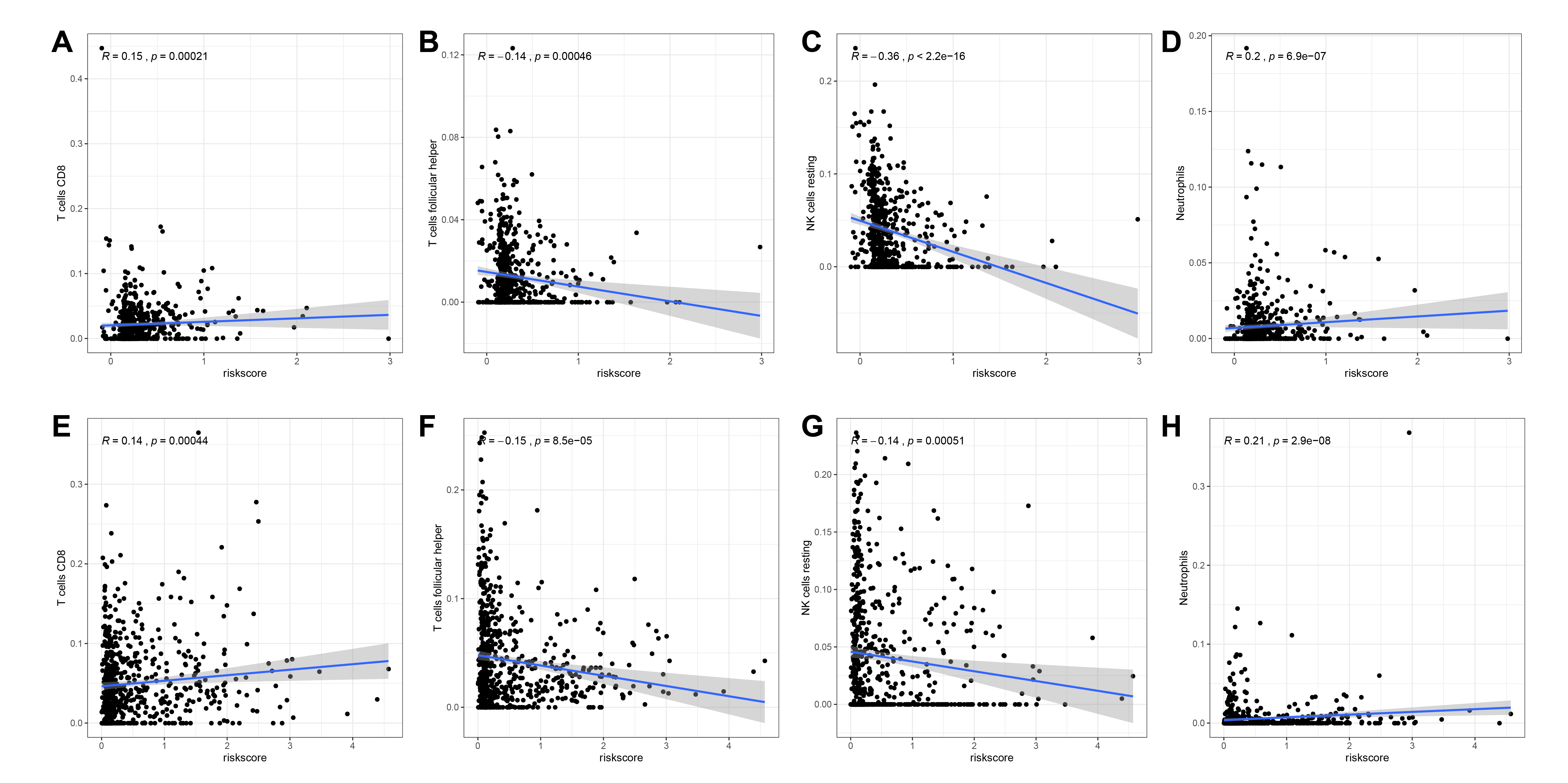

Supplement: Supplementary File S5 — Correlation between the immune cell abundance and the PROMISE risk score. (A–D) Scatter plot of the Spearman correlation analysis in TCGA glioma samples. (E–H) Scatter plot of the Spearman correlation analysis in CGGA Glioma samples. [file Image_2.tif]
